# Cost- Effectiveness of Mammography Screening Program in a Resource-Limited Post-Soviet Country of Kazakhstan

**DOI:** 10.31557/APJCP.2019.20.10.3153

**Published:** 2019

**Authors:** Islam Salikhanov, Byron Crape, Peter Howie

**Affiliations:** *Nazarbayev University, School of Medicine, Nur-Sultan, Kazakhstan.*

**Keywords:** Breast cancer, mammography, screening, cost-effectiveness analysis, benefit, cost analysis

## Abstract

**Objectives:**

To conduct cost effectiveness and benefit-cost analyses of the organized mammography-screening program in the Republic of Kazakhstan comparing women who developed breast cancer in screened and unscreened scenario.

**Methods:**

389,352 screened women were included in the study. Among these, 895 women were further diagnosed with breast cancer. Outcomes measures include life years saved, quality-adjusted life years, incremental cost-effectiveness ratio, and value of statistical life year. Sensitivity analyses were performed to assess uncertainty.

**Results:**

Compared to no screening scenario, an organized mammography yielded an additional 1,253 life years and 790 quality-adjusted life years in 2016. The incremental cost-effectiveness ratio was equal to 3,157 USD per one QALY saved, which is two times less than the GDP per capita in Kazakhstan in 2016. Sensitivity analysis showed that the mammography remains cost-effective in the majority of the scenarios.

**Conclusion:**

Mammography screening in Kazakhstan was found to be highly cost-effective, associated with treatment cost savings, and can be an efficient use of limited resources in Kazakhstan.

## Introduction

Breast cancer is the most commonly diagnosed type of cancer in a resource-limited post-Soviet country of Kazakhstan (Baizhumanova and Sakamoto, 2010; Toleutay et al., 2013). In some regions of Kazakhstan, incidence rates of breast cancer have reached 274 per 100,000 (Bilyalova et al., 2012). In 2014, breast cancer accounted for 17% of all deaths from cancer in Kazakhstan (http://www.who.int/cancer/country-profiles/kaz_en.pdf).

In Kazakhstan, national mammography screening program was introduced in 2008. It is a biennial program, which targets women aged 50, 52, 54, 56, 58, and 60 (Beysebayev E. et al., 2015). 

During 2016, the mammography-screening program covered 389,352 women and identified 895 new cases of breast cancer, which amounted to 0.2% of the total number of screened women.

Compared to the five-year survival rate of women who underwent mammography, which was 98.3%, the survival rates of women with breast cancer who underwent clinical examination and self-detection were 94.3%, and 84.8%, respectively (Kawai et al., 2009). It is projected that further implementation of the nation-wide mammography-screening program in Kazakhstan would obtain an additional health benefits in breast cancer outcomes at a lower cost. However, currently there is absence of evidence regarding the health and economic outcomes of the breast cancer-screening program in Kazakhstan. To the best of our knowledge, this is the first cost-effectiveness analysis of breast cancer screening program in the post-Soviet region. 

The main objective of this research is not only to assess the health outcomes associated with screening, but also to evaluate the economic aspects and financial justification of using it in a resource-constricted country. 

## Materials and Methods

*Research design*

The research design was a cross-sectional based on data for the year 2016 utilizing cost-benefit analysis and cost-utility analysis were conducted to assess the economic feasibility of the mammography-screening program in the Republic of Kazakhstan from the perspective of the Ministry of Health. The Ministry of Health funds and controls the national screening program. Cost-benefit analysis was utilized as it allows costs to be justified not only in terms of health effects but also in monetary benefits, thus, allowing us to see the effectiveness of the use of an allocated budget (Sartori et al., 2014). 

*Research Outcomes*

To conduct a comprehensive health economics analysis we used several measurements of health and financial outcomes of the breast cancer screening. These include life years saved, quality-adjusted life years (QALYs), incremental cost-effectiveness ratio, and value of a statistical life (Blomqvist, 2002).

A measurement that is used in cost-benefit analysis is a value of a statistical life, which represents the marginal rate of substitution between income and mortality risk (Blomqvist et al., 2002). The value of a statistical life can be used as a measure of the societal cost and the social impact of the screening program. Therefore, using indicators of the cost of screening and treatment, and the number of QALYs gained, we were able to calculate the incremental cost-effectiveness ratio (Shaun et al., 2017). Based on the data availability, the analysis is based on the performance of the mammography-screening program in the year 2016.

*Screening Group*

Screening group includes 895 new cases of breast cancer that the mammography-screening program identified in 2016. Life years saved and QALYs saved in this group were calculated utilizing the breast cancer stage distribution for women who participated in mammography screening and, as a result of referral to a physician, had been diagnosed with breast cancer.

*Control group*

The control group is based on the breast cancer stage distribution of women who had never received mammography screening and, thus had a different breast cancer stage distribution as never- screened women. Table 3 demonstrates the five-year survival and stage distribution of women with breast cancer in 2016 in Kazakhstan. The data on stage distribution of screened and unscreened women had been provided by the Center of Oncology in Nur-Sultan. 

*Costs and discount rate*

Relevant data on costs were provided by the Center of Oncology in Nur-Sultan. As there is no data about the discount rate for Kazakhstan, we applied 4.8% social rate of time preferences for healthcare used in Russian Federation, as economies of our countries are highly integrated with each other (Kossova and Sheluntcova, 2016). The dollar to tenge exchange rate for the June 2016 was utilized at USD 1= 336 KZ tenge.

On average, mammography per one woman in Kazakhstan costs 14 USD (5,000 tenge). The total cost of the mammography-screening program includes the expenses for equipment, technical support, staff education, software, infrastructure and salaries. In total, the annual cost of the mammography-screening program in 2016 was 5,450,928 USD, which resulted from screening of 389,352 women. According to the decree of the government, oncological services in Kazakhstan are financed by the state budget. In Kazakhstan, costs associated with treatment of breast cancer vary drastically depending on the stage of the tumor. The average price per clinical examination is about 9 USD. Costs of treatment include chemotherapy, surgery, and radiation. The cost of one course of chemotherapy in Kazakhstan is about 500 USD, while women with advanced stages of breast cancer need at least 4 such courses. Additionally, surgical treatment of breast cancer costs about 1,500 USD. Radiation therapy is considered the most expensive as it costs the government about 30,000 USD per woman. Therefore, the cost of treatment for a woman with stage I breast cancer is about 5,000 USD; treatment of stage II breast cancer costs around 30,000 USD, and treatment of stage III and IV breast cancer costs from 36,000 USD to 40,000 USD, respectively. 

*Five-year survival analysis*

A key assumption about mammography is that it identifies a higher proportion of breast cancer at earlier stages that would have otherwise progressed to an advanced stage. Based on five-year survival rates and breast cancer stage distribution, we analyzed breast cancer mortality rates among 895 screened women and compared them with mortality rates in the unscreened control group. The data on stage distribution was provided by the Oncology Center of Nur-Sultan. 

Understanding the additional life years given to women by early detection of breast cancer is essential as substantial finances are invested to provide mammography to the population. Median survival rates were applied to estimate the difference of life years saved due to screening versus no screening.

QALY estimate has been used in our analysis as it allows adjusting for the quality of life of women diagnosed with breast cancer (Robberstad, 2005). To estimate the number of QALYs gained by the mammography-screening program among women aged 50, 52, 54, 56, 58, 60 with breast cancer, it is essential to estimate their life expectancy, adjust for quality of life, and to compare it with QALYs in no screening scenario based on the difference in stage distribution between screened and unscreened scenario. 

*Life expectancy*

According to the WHO life tables, the additional expected life of women aged 50-54 years in Kazakhstan is around 28 years, and the additional expected life of women aged 55-59 is 23.7 years (http://apps.who.int/gho/data/?theme=main&vid=60840). Weighted average life expectancy of women aged 50-60 is calculated at 26 years. Such an adjustment is necessary for our analysis as it demonstrates that the mammography-screening program can save additional life years, which significantly changes QALYs. Table 2 provides the data on life expectancy of women with different stages of breast cancer by age. 

*Quality of life*

Quality of life estimate is utilized as it adjust the number of saved life years for the quality of life of women with different stages of breast cancer (Mosteller and Falotico-Taylor, 1989). Because of illnesses related to aging, the normal quality of life coefficient of women aged 50-60 years was previously computed to be 76% (Stout and Rosenberg, et al., 2006). Table 2 shows quality of life coefficients for different stages of breast cancer. Quality of life of women with stage I of breast cancer is assumed to be 90% of the estimate of quality of life of a normal person. Moreover, quality of life for stage II of breast cancer is 75% of the normal state, while it is less than 60% for women with stage III-IV of breast cancer and distant metastasis (Stout et al., 2006).

To assess the quality of life of women after remission, the natural decrease of the quality of life index needs to be taken into account. Thereby, quality of life coefficient of healthy women aged 50-60 is about 76% of quality of life of a healthy women aged 25-30. Whereas for women aged 61-70, quality of life coefficient is equal to 74%, and for women aged 71-80 it is 70% (Stout et al., 2006).

Incremental Cost-Effectiveness Ratio To assess the cost of the mammography-screening program we incorporated the total cost of screening itself and cost of treatment of 895 diseased women. The incremental cost-effectiveness ratio has been applied as a measure used in benefit-cost and cost-utility analysis of health care interventions (Bang and Zhao, 2012). The incremental cost-effectiveness ratio is defined by dividing the difference between the costs of two interventions by the difference in their health outcomes (QALYs gained). The incremental cost-effectiveness ratio represents the average cost associated with one extra unit of the measure of effect (USD per QALY). 

The incremental cost-effectiveness ratio (ICER) was estimated as: ICER=(C1-C0) / (E1-E0) 

Where C1 and E1 are cost and effect in the intervention group (screened) and where C0 and E0 are cost and effect in the control group (non-screened) (Bang and Zhao, 2012).

The value of a statistical life estimate has been utilized as it indicates how much an individual is willing to pay for a small reduction in the risk of death (Blomqvist, 2002; Tekeşin and Ara, 2014). 

The value of a statistical life in Kazakhstan was estimated using the following formula (Robinson et al., 2019): 

VSL target = VSL base * (Income target / Income base)^elasticity^

Since changes in income leads to proportionate changes in the value of a statistical life, an elasticity equal to “1” was used. The value of a statistical life is usually estimated at the average age, which is 29.3 years for Kazakhstan (Robberstad, 2005).

*Sensitivity Analysis*

We conducted 25 different one-way sensitivity analyses. First sensitivity analysis is based on the coverage rate observed under the mammography-screening program. For this purpose, we used 30%, 50%, 70%, 90% and 100% coverage rates to assess how such diverse screening scenarios can affect our outcomes. The upper limit of cost-effectiveness is triple the GDP per capita threshold, which in 2016 was USD 22,530 (www.data.worldbank.org). Additionally, we assessed how different value of a statistical life can change the benefits of mammography. We used such values of a statistical life as USD 500,000, USD 1,000,000, USD 1,200,000, and USD 2,000,000. We also conducted sensitivity analyses based on the QALYs gained by mammography and then assessed the impact of different discount rates on the financial outcomes. Therefore, these sensitivity analyses considered scenarios where the benefits of the screening are much smaller than what was shown in our base case analysis. In addition, we used one-way deterministic sensitivity analysis varying the average cost of treatment from USD 10,000 to USD 30,000.

## Results

*Health outcomes and stage distribution*

During 2016, the mammography-screening program identified 895 breast cancer cases. Out of these 60% were diagnosed with stage I; 25% stage II; 15% stage III; and 5% stage IV. Among women who did not undergo screening, 30% developed stage I; 30% stage II; 25% stage III; and 15% stage IV. Table 2 provides information on survival rates and stage distribution observed among the screened and unscreened groups. 

Table 2 shows that the five-year survival rates for the mammography-screened group were about 80%, while for among unscreened women, it was estimated to be 65%. Women with breast cancer who had mammography screening was associated with 15% reduction in breast cancer mortality rates. 

Table 1 demonstrates that treatment of 895 mammography-screened women saved 6,936 life years. Whereas treatment of 895 non-screened women 5,683 life years. Hence, in 2016, the mammography screening program gained additional 1,253 life years.

The average discounted cost of treatment per screened woman with breast cancer in 2016 was equal to 12,917 USD, while the average discounted cost of treatment for an unscreened woman with breast cancer in the same year was equal to 16,221 USD. 

Therefore, the total cost of screening of 389,352 women and treatment of 895 mammography-screened women with breast cancer was 17,012,476 USD, while an in the unscreened group scenario of the same number of women the cost of treatment was estimated to be around 14,518,256 USD.

Table 5 shows that in 2016 the total number of QALYs due to screening in 2016 was amounted to 15,274 for the 895 women. Without screening, the number of QALYs breast cancer treatment of was 14,484 QALYs. Therefore, in 2016 mammography screening gained additional 790 QALYs. 

Table 4 summarized the value of a statistical life estimate for Kazakhstan in 2016. We used value of a statistical life and income from Russia as the base. Average value of a statistical life in Russia is equal to 1.6 million USD (Stout et al., 2006). Average monthly income in Kazakhstan in 2016 amounted to 450 USD, while in Russia it was around 600 USD. 

Therefore: VSL_Kz_=VSL_Rus_*(Income K_z_/Income _Rus_) 

VSL_Kz_=1,600 000*(450/600) = 1,200 000 USD

The total life expectancy of women aged 50-60 is about 76 years, 46 additional years beyond the mean average age. With a 4.8% social discount rate over 46 years, the average value of a statistical life year in Kazakhstan is estimated to be 65,018 USD. Therefore, we are able conclude that screening saved 51,364,220 USD, since the mammography program saved 790 quality-adjusted life years in a year 2016. Thus, with an additional budget of 2,494,220 USD in 2016, mammography saved the value of a statistical life estimated to be around 51,364,220 US dollars. 

To assess incremental cost-effectiveness ratio we need to estimate the difference between costs per screened and unscreened women who had breast cancer. Total treatment cost of screened woman includes 5,450,928 USD for screening and 10,721,883 USD for treatment. Therefore, the total cost amounted to 16,172,811 USD. 

Table 5 demonstrates that the cost of treatment of the unscreened group in 2016 was 14,518,256 USD. Thus, the mammography-screening program gained 790 QALYs with an additional budget of 2,494,220 USD. Therefore, the incremental cost-effectiveness ratio of the mammography-screening program in 2016 amounted to 3,157 USD per one QALY gained, which is less than the national annual GDP per capita in Kazakhstan, which in 2016 estimated to be 7,510 USD (www.data.worldbank.org/). 

*Sensitivity Analysis*

Among 1,506 patients diagnosed with breast cancer in 2016, 895 underwent mammography and as a result were referred to a physician who diagnosed them with breast cancer. Out of the remaining 611 breast cancer patients, 183 (based on 83% sensitivity for mammography screening (Hofvind et al., 2012; Jacobsen et al., 2015; Ferrini et al., 1996) had participated in mammography but were not identified and referred for diagnostic testing (false negatives). Therefore, women who had false negative results develop advanced stages of breast cancer like women who have never been screened before.

**Figure 1:**
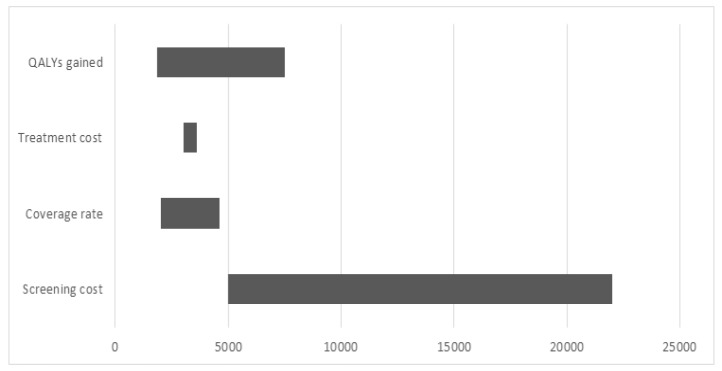
One-Way Sensitivity Analysis Utilizing Coverage, QALYs Gained, and Costs of Screening and Treatment. Tornado Diagram. (MS Excel). The diagram summarizes the results of one-way sensitivity analysis. The x-axis represents the incremental cost-effectiveness ratio (USD) per QALY gained by the mammography screening program over no screening. The y-axis represents the parameters that were changed and affected the incremental cost-effectiveness ratio. Under 100% coverage of the target age group scenario, the number of screened women rises from 389,352 to almost 550,000, what increases the cost of the breast screening from 5,450,928 to 7,700,000 USD [37]. Tables 6 shows that in this case, mammography saves 2,189 QALYs with the incremental cost-effectiveness ratio equal to 1,012 USD per one QALY. Thus, higher coverage of the target age group by the screening would be highly cost-effective [21]

To assess the impact of different variables on health and financial outcomes we conducted 25 one-way sensitivity analyses. Even with the attendance rate equal to 30%, the incremental cost-effectiveness ratio is still lower than the GDP per capita threshold. Higher coverage rate of mammography resulted in lower incremental cost-effectiveness ratio. Changing average cost of treatment did not affect the domination of mammography. In addition, as the cost of treatment and the number of life years saved rise, the incremental cost-effectiveness ratio decreases. The sensitivity analysis using coverage rate, QALYs gained, treatment and screening costs is summarized in Figure 1.

Variations of gained QALYs also affected the outcome. The incremental cost-effectiveness ratio would be higher than the GDP per capita threshold if the number of gained QALYs is below 380 QALYs. The greatest impact on the incremental cost-effectiveness ratio was observed with the rise of the average cost of screening per one woman. The range of variation in the cost of one utilized mammogram is 14 to 50 USD, where the cost of mammography per woman in KZ is estimated as USD 14 and USD 50 is the estimated cost of mammography per woman in some European countries (Gad et al., 2011). Rising cost of one mammogram up to 50 USD moves incremental cost-effectiveness ratio to the triple GDP per capita threshold making it not to be cost-effective.

European cost was applied in the sensitivity analysis as European guidelines are being used for implementation of the screening program in Kazakhstan. Changes in the stage distribution of breast cancer cases from an earlier to an advanced stages also increased the incremental cost-effectiveness ratio, decreased the number of life years saved, and may result in dominance of the no screening scenario. Despite variations in the coverage rates, screening dominated over no screening scenario. If we restrict the value of a statistical life in Kazakhstan to 100,000 USD, we see a decrease in the benefits of the mammography-screening program. However, such a restriction does not lead to financial ineffectiveness of the mammography-screening program. Finally, Table 7 demonstrates the sensitivity analysis using 5 parameters.

At the same time, based on a willingness-to-pay a threshold of triple the GDP, a total of USD 22,530 per life-year-gained in 2016, the mammography screening is cost-effective (Schiller-Fruehwirth et al., 2017). However, as the willingness-to-pay is associated with income and circumstances, the cost-effectiveness threshold based on willingness-to-pay was not utilized in this study (Wagnera et al., 2000).

## Discussion

To the best of our knowledge, this is the first published health economic analysis of the breast cancer-screening program in the post-Soviet region. Our study found that in 2016 the mammography-screening program in Kazakhstan was cost-effective with the incremental cost-effectiveness ratio equaled to USD 3,157. Mammography screening has proven to be effective in several developed countries (Nyström et al., 1993; Fracheboud et al., 2003; Fruehwirth et al., 2017; Arrospide et al., 2016). However, the cost-effectiveness of mammography screening programs in post-Soviet economic transitional countries such as Kazakhstan is absent. This analysis supports a patient-centered decision-making to judge the benefits of the mammography screening while taking into consideration its financial and non-financial burden. Countries with established mammography screening programs are more likely to identify cases of breast cancer at an earlier stages compared to countries that did not introduce national screening (Vieira et al., 2017). 

**Table 1:** Comparison of Saved Life Years between Screening and No Screening Scenario Based on Median Survival and Stage Distribution of Breast Cancer in Kazakhstan In 2016

Stage	Median Survival Rates for each stage (years)	Screening Scenario		No screening Scenario	
		Number of patients	Life Years Saved	Number of patients	Life Years Saved
1st	9	537	4833	268	2416
2nd	7	224	1566	268	1879
3rd	5	90	448	224	1119
4th	2	45	89	135	269
Total			6,936		5,683

**Table 2:** Quality of Life and Life Expectancy of Women with Breast Cancer in Kazakhstan in 2016. Life expectancy estimates have been assessed according to the WHO life tables. Quality of life coefficients have been obtained from literature review (Natasha K. Stout, 2006)

	Health State					Life expectancy
Age	Healthy	Stage I	Stage II	Stage III	Stage IV	
50-54	0.78	0.696	0.644	0.598	0.536	28
55-60	0.747	0.694	0.636	0.567	0.474	23.7

**Table 3:** Five-Year Survival Difference between Screened and Unscreened Women with Breast Cancer Based on Stage Distribution in 2016 in Kazakhstan

Stage	Five-year survival	Distribution Screened	Distribution	Cohort five-year survival		Difference
			Unscreened	Screening	No screening	
Stage I	95%	60%	30%	0.57	0.285	
Stage II	70%	25%	30%	0.175	0.21	
Stage III	50%	10%	25%	0.05	0.125	
Stage IV	22%	5%	15%	0.011	0.033	
Total				0.806	0.653	0.153

Note: Based on five-year survival rates and stage distribution in both cohorts we estimated expected difference in survival rates between two groups

**Table 4:** Value of a Statistical Life Estimate for Kazakhstan in 2016

Value of a statistical life in Kazakhstan	$1,200,000
Value of a statistical life year in Kazakhstan	$65,018
Value of a statistical life saved by the mammography screening program	$51,364,220

**Table 5:** Incremental Cost-Effectiveness Ratio of the Mammography Screening Program with 4.8% Discount Rate (Monetary Values in USD Millions) in Screnning and No Screening Scenario in Kazakshtan in 2016

	Screened cohort	Unscreened cohort	Difference
Total costs	17	14.5	2.5
Screening costs	5.4	0	5.4
Diagnosis and treatment costs	11.6	14.5	-2.9
QALYs gained	15,274	14,484	790
Incremental cost-effectiveness ratio (USD/QALY)			3,157

**Table 6:** The Costs of Mammography under 100% Coverage Scenario vs. Costs of Mammography under no Screening Scenario in USD in Kazakhstan in 2016

	100% coverage	No coverage
Treatment cost	21,442,220	26,926,860
Screening cost	7,700,000	0
Total cost	29,142,220	26,926,860
Cost difference	2,215,360	
QALYs saved	2,189	
Incremental cost-effectiveness ratio	1,012	

**Table 7:** Results of One-Way Sensitivity Analysis. The upper limit of cost-effectiveness is triple GDP per capita threshold, which in 2016 was USD 22,530

Input parameter	Range	Influence on model
Coverage	30-100%	The lower is the coverage - the higher is the ICER. Within 30-100% coverage, the screening is cost-effective.
Cost of mammography per woman	USD 14-50	The increase of cost of 1 mammogram to USD 50 increases the ICER and makes the program not cost-effective
Treatment cost	USD 10,000 – 30,000	Increase in costs of treatment decreases the ICER.
Cases detected	350-1,100	No change of cost-effectiveness
QALYs gained	300 – 2,000	The ICER is higher than the GDP per capita if the number of gained QALYs is below 380 QALYs, thus, not-cost-effective.

Recent studies have shown that the sensitivity of mammography increases with age (Peer et al., 1996; Kerlikowske et al., 1996). International experience shows that the biennial mammography-screening has approximately 83% sensitivity and 90% specificity (Hofvind et al., 2012; Jacobsen et al., 2015; Ferrini et al., 1996). In addition, some research demonstrated that the breast screening sensitivity is predominantly dependent on breast tissue density (Kerlikowske et al., 1996). One of the indicators of the effectiveness of screening is the constant decrease in the rates of breast cancer cases at advanced stages. In Kazakhstan, mammography led to a shift in the stage distribution of breast cancer in a way that a smaller quantity of cases were diagnosed at stages III and IV. Concurrently, there has been a major increase in the number of breast cancer at Stage I. We conducted an extensive sensitivity analyses and found that in the majority of scenarios the incremental cost-effectiveness ratio would stay below the cost-effectiveness threshold. Other middle income countries, such as Kazakhstan, that also conducted cost-effectiveness analysis of mammography screening programs include Iran, Turkey, India and China. 

The results of mammography screening in Iranian women aged 40-70 years found that the incremental cost effectiveness ratio was USD 37,350 per QALY gained, considered cost effective (Haghighat et al., 2016). Okonkwo et al., (2008) reported the cost of mammography among Indian women aged 40-69 was USD 1,341 per life-year-gained. also considered cost effective. A study conducted at the Bahçeşehir Mammography Screening Project in Turkey found that biennial mammography screening is economically feasible, with costs of USD 2,423 per saved life year (Özmen et al., 2017). However, Wong et al. found the cost of biennial mammography for women in Hong Kong for ages 40 to 69 years of USD 61,600 per QALY, was not cost effective based on a USD 50,000 threshold (Wong et al., 2007). All above-mentioned breast screening programs, including all of those who were cost effective are biennial. If annual mammography screening was implemented, costs increase substantially and may not meet the WHO criteria for cost-effectiveness (Okonkwo et al., 2008).

Life years saved is a relatively simple and transparent measurement of population health. Our analysis shows that mammography saved 1,253 life years. Therefore, to increase the number of life years gained due to mammography, its coverage rate should be considered among our most central public health concerns. 

However, the estimated life years saved is often criticized as it ignores changes in health state in comparison with QALYs (Robberstad et al., 2005). Our research shows that in 2016 mammography saved additional 790 QALYs per 895-screened women, compared to the no screening scenario. According to the WHO policy, an incremental cost-effectiveness ratio less than the national annual GDP per capita is considered highly cost–effective. Incremental cost-effectiveness ratio of the mammography-screening program in Kazakhstan showed that the mammography-screening program in Kazakhstan is highly cost effective. 

As sensitivity of screenings is dependent on choice of screening equipment, the “Mammonat 3000 NOVA” mammograph, which is the commonly used equipment for breast-screening in Kazakhstan, has a population-based sensitivity of 80% and specificity of 83%. 

The chance of getting a false positive from the first mammogram ranges from 7 to 12 percent, depending on the age the woman, as younger women are more likely to have false positive results (Nelson et al., 2016). After 10 mammograms, the chance of getting a false positive result is approximately 50 to 60 percent (Hubbard et al., 2011; Marmot et al., 2013; Nelson et al, 2016). As case detection rate is a ratio of the number of detected cases to the number of the total incident cases in a given year, the case detection rate of breast cancer was 60%, based on 895 detected cases out of 1,506 cases of breast cancer in Kazakhstan in 2016 (Wallis et al., 2007).

Our study indicates that despite the contextually high cost of the mammography-screening program, it leads to substantial savings because of its ability to detect breast cancer at an early stage leading to saving on breast cancer treatment. At the same time, mammography affects women’s personal quality of life by early diagnosis and prevention of the development of breast cancer to advanced stages (Al-Naggar et al., 2011).

We used the value of a statistical life to assess the social impact of screening. The value of a statistical life estimate showed that the mammography-screening program could lead to substantial social cost savings.

The consequences of late diagnosis of breast cancer are lower survival rates, higher morbidity rates and higher costs of care, resulting in disability and avoidable deaths. Early mammography screening is a vital public health strategy in all settings since it improves outcomes by identifying cancer at the earliest stages (www.who.int/cancer/publications/mammography_screening/en/). 

We performed sensitivity analyses, which shows that mammography screening in Kazakhstan is cost effective in the majority of scenarios and remains below the GDP per capita threshold. After the program was launched, overall breast cancer mortality rates has constantly decreased. This is similar to developed countries expenses with established mammography program. For example, in Sweden mammography led to 31% decline of mortality rates from breast cancer, while in Switzerland death rates from breast cancer dropped by 19% (Autier et al., 2012; Gelder et al., 2009). Australia’s mammography screening reduced mortality rates from breast cancer by 28% (Budd et al., 2010). Such decrease in mortality rates may also be expected in Kazakhstan under higher screening rates.

Our analysis has some limitations that should be considered. We analyzed screening from the perspective of the Ministry of Health so our research does not take into account personal patient costs of breast cancer, such as salary loss, costs of transportation from rural areas, costs of medicine, etc. breast cancer (Broekx et al., 2011). In addition, screening itself may cause non-monetary harms like over-diagnosis or false positive results what may lead to unnecessary treatment and radiation (Morris et al., 2015; DeFrank et al., 2012). Estimating such harms would contribute to a more comprehensive analysis of breast cancer screening program. Moreover, the lack of some official statistics in the Ministry of Health required utilization of some information from published findings from similar health systems in other countries. 

Our health economics analyses indicates that the mammography-screening program in Kazakhstan is cost-effective. There are no international peer-reviewed publications in post-Soviet economic transitional countries regarding cost-effectiveness of the mammography-screening program. Future analyses can contribute by integrating personal expenditures and costs of over-diagnosis and false-positive results into analyses of mammography screening programs. 

Screening programs should be introduced only when their effectiveness has been proved and resources are adequate to cover the target group (http://www.who.int/cancer/detection/en/).

Finally, when planned efficiently, properly financed and implemented, screening can reduce mortality rates and the risk of developing advanced stages of breast cancer (Cox et al., 2008). 
